# Keratinocyte-Targeted Overexpression of the Glucocorticoid Receptor Delays Cutaneous Wound Healing

**DOI:** 10.1371/journal.pone.0029701

**Published:** 2012-01-03

**Authors:** Ana Sanchis, Lorena Alba, Víctor Latorre, Lisa M. Sevilla, Paloma Pérez

**Affiliations:** Department of Pathology and Cell and Molecular Therapy, Instituto de Biomedicina de Valencia (IBV)-Consejo Superior de Investigaciones Científicas (CSIC), Valencia, Spain; University Hospital Hamburg-Eppendorf, Germany

## Abstract

Delayed wound healing is one of the most common secondary adverse effects associated to the therapeutic use of glucocorticoid (GC) analogs, which act through the ligand-dependent transcription factor GC-receptor (GR). GR function is exerted through DNA-binding-dependent and –independent mechanisms, classically referred to as transactivation (TA) and transrepression (TR). Currently both TA and TR are thought to contribute to the therapeutical effects mediated by GR; however their relative contribution to unwanted side effects such as delayed wound healing is unknown. We evaluated skin wound healing in transgenic mice with keratinocyte-restricted expression of either wild type GR or a mutant GR that is TA-defective but efficient in TR (K5-GR and K5-GR-TR mice, respectively). Our data show that at days (d) 4 and 8 following wounding, healing in K5-GR mice was delayed relative to WT, with reduced recruitment of granulocytes and macrophages and diminished *TNF-α* and *IL-1β* expression. *TGF-β*1 and *Kgf* expression was repressed in K5-GR skin whereas *TGF-β*3 was up-regulated. The re-epithelialization rate was reduced in K5-GR relative to WT, as was formation of granulation tissue. In contrast, K5-GR-TR mice showed delays in healing at d4 but re-established the skin breach at d8 concomitant with decreased repression of pro-inflammatory cytokines and growth factors relative to K5-GR mice. Keratinocytes from both transgenic mice closed *in vitro* wounds slower relative to WT, consistent with the *in vivo* defects in cell migration. Overall, the delay in the early stages of wound healing in both transgenic models is similar to that elicited by systemic treatment with dexamethasone. Wound responses in the transgenic keratinocytes correlated with reduced ERK activity both *in vivo* and *in vitro*. We conclude that the TR function of GR is sufficient for negatively regulating early stages of wound closure, while TA by GR is required for delaying later stages of healing.

## Introduction

Glucocorticoid (GC) derivatives are widely used as therapeutic agents in acute and chronic inflammatory diseases due to their anti-inflammatory properties [1]. However, unwanted side-effects, such as delayed wound healing, are very common, limiting their long-term use [1]–[3]. GCs act through the ubiquitously expressed intracellular GC receptor (GR), encoded by a single gene in humans and mice called *Nr3c1*. *Nr3c1* transcripts undergo both alternative splicing and alternative translational initiation, resulting in multiple GR protein isoforms [4]. GRα, which we will refer to as GR, is the predominant mRNA isoform in most cell types and its protein products are able to bind endogenous or synthetic ligands. After binding GCs, GR dissociates from cytoplasmic complexes, dimerizes and translocates to the nucleus, where it modulates gene transcription [5]. GR monomers or dimers can bind to GC-response elements or GREs located in target genes [6]. Besides DNA-binding-dependent transcriptional regulation, GR also regulates gene expression by interfering with other transcription factors, such as NF-κB, AP-1 or STATs, without direct binding to DNA [7], [8].

The mechanisms of GR-mediated regulation are classically referred to as transactivation (TA; dimerization- and DNA-binding-dependent) and transrepression (TR; DNA-binding-independent). The genetic dissection of TA and TR functions has contributed to a better understanding of the mechanisms of GR action. A great deal of work has been performed with GR^dim/dim^ mice that harbor a point mutation (A458T) in the DNA binding domain of GR, abrogating dimerization-dependent TA [9]. In contrast to GR^−/−^ mice, which die perinatally from respiratory distress, GR^dim/dim^ mice survive, thus allowing studies in adult animals [10], [11]. Other GR mutants with compromised TA that retain their capacity for TR have been reported, including P493R/A494S [12], here referred to as GR-TR. Classically, the therapeutical actions of GR have been ascribed to TR and the adverse side-effects have been linked to TA, however, the scenario is far more complex. More recent studies demonstrate that both TA and TR contribute to GR's anti-inflammatory effects, for instance, through the induction of genes such as *Mkp-1* and *Ikba* [13], [14].

Cutaneous wound healing is a complex process aimed to repair tissue damage and restore skin homeostasis. There are three phases in wound healing that require a coordinated interaction among diverse cell types with precise kinetics: inflammation, re-epithelialization and tissue remodeling [15], [16]. In all phases of wound healing, growth factors and cytokines play a major role [16]. The first phase of wound repair is inflammation, and is characterized by platelet aggregation followed by recruitment of leukocytes including macrophages and neutrophils at to the injury site. The release of cytokines and growth factors amplifies the inflammatory response and initiates the formation of the granulation tissue. This phase is key for subsequent re-epithelialization and remodeling and thus constitutes a target for modulating the outcome of the healing response. Re-epithelialization requires both proliferation and migration of keratinocytes, and is normally accompanied by formation of new granulation tissue. After re-epithelialization is completed, keratinocytes must stratify and differentiate to restore the barrier. The granulation tissue has to be replaced by extracellular matrix proteins such as collagen and elastin to achieve complete tissue remodeling.

Previous work has evaluated the mechanisms by which GR action inhibits healing by using rodent models treated with GC analogs, mainly Dexamethasone (Dex) [17]. In this work, we assessed the consequences of constitutive, keratinocyte-specific expression of the wt GR or the GR-TR mutant on the wound healing process. For this purpose, we have used the transgenic mouse lines K5-GR and K5-GR-TR, in which both transgenes are under the control of the same tissue-specific promoter achieving similar expression levels of the WT and mutant GR proteins [18], [19].

Altogether, our data indicate that keratinocyte-targeted overexpression of either GR or GR-TR was sufficient to repress re-epithelialization at early timepoints following wounding. Both transgenes inhibited keratinocyte proliferation and migration to a similar extent relative to controls. However, the repression of several cytokines and growth factors associated with wound repair was more pronounced in K5-GR mice than in K5-GR-TR mice. Despite the initial delay in re-epithelialization, K5-GR-TR, but not K5-GR, mice were eventually able to heal wounds with similar kinetics as WT. *In vitro* wound scratch assays using cultured keratinocytes isolated from K5-GR and K5-GR-TR mice recapitulated the *in vivo* observations.

## Results

**Figure 1:**
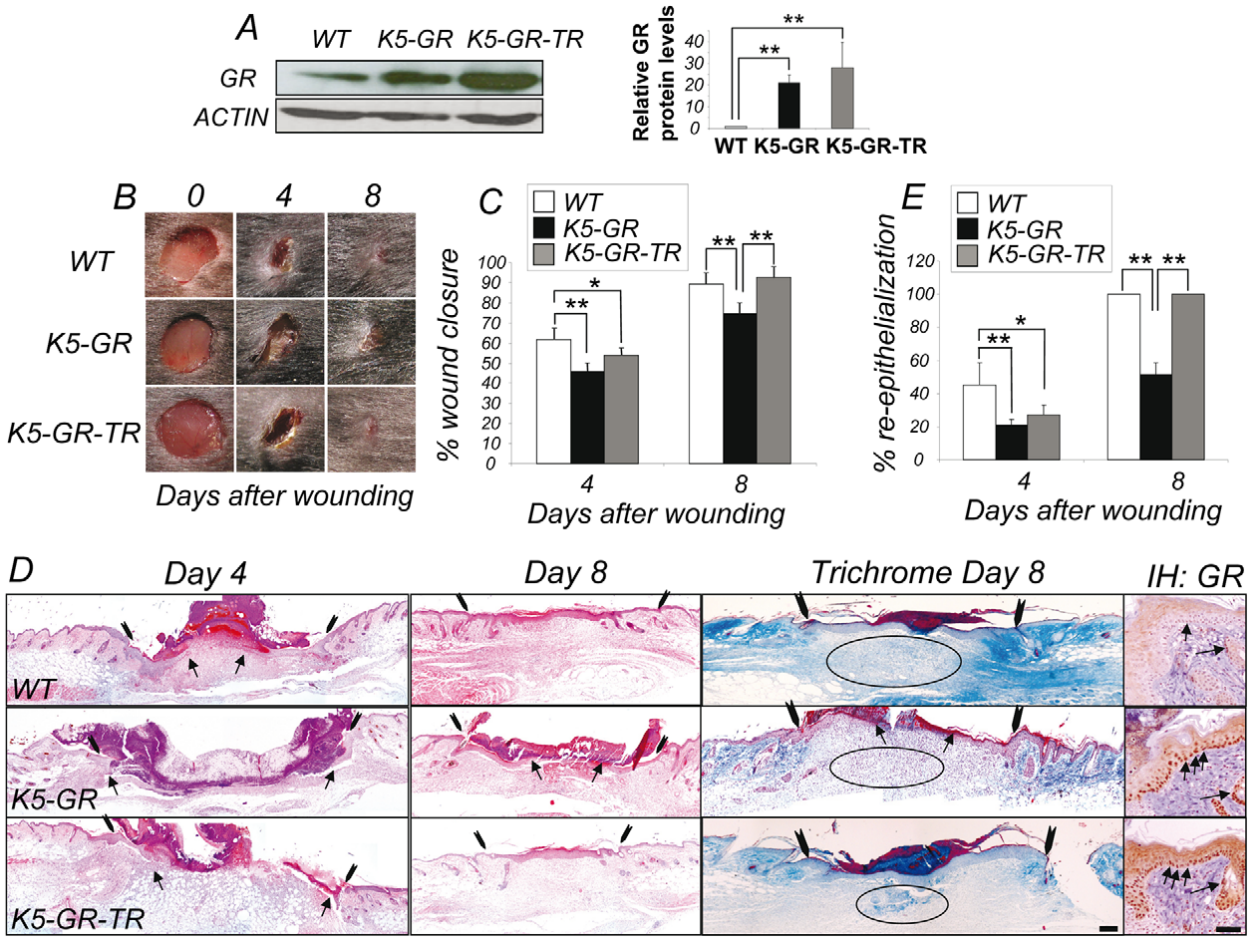
*In vivo* overexpression of GR and GR-TR in keratinocytes delays wound healing with different kinetics of repair. A) Similar transgene expression in K5-GR and K5-GR-TR mice. Relative protein levels of the transgene *vs* endogenous GR were determined by immunoblotting using an antibody that recognizes both mouse (endogenous) and rat (transgene) GR. Actin was used as a loading control. A representative panel of three independent experiments and quantitation of three independent experiments is shown. Statistical significance was assessed by ANOVA (**p<0.01). B) Macroscopic changes in full-thickness cutaneous wounds. The wound sites were photographed at the time indicated after wounding. Representative results from 12 mice of each genotype are shown. C) The percentage of wound closure area at each time point was estimated relative to the original wound area. Values represent mean ± SD (*n* = 12 for all groups). Statistical significance was determined using ANOVA (*p<0.05, **p<0.01). D) Histological evaluation of skin wounds in WT, K5-GR and K5-GR-TR mice at d4 and 8 after injury. Arrowheads and arrows indicate original wound edges and re-epithelialized edges, respectively. Trichrome staining shows distinct collagen deposition in the dermis at d8 in the indicated genotypes. Immunostaining using an anti-GR specific antibody shows GR expression and localization in WT *vs* transgenic mice (IH: GR). Note the nuclear localization of GR in both transgenic skin samples. Hematoxylin/eosin and Trichrome staining, Bars: 100 µm; IH: GR, Bar: 50 µm. E) The percentage of re-epithelialization was evaluated in WT, K5-GR and K5-GR-TR mice at d4 and d8 after wounding. All values represent the mean ±SD (*n* = 12 for all groups). Statistical significance was estimated using ANOVA (*p<0.05, **p<0.01).

We analyzed the kinetics of healing in K5-GR and K5-GR-TR mice relative to WT littermates after excision wounds were made on dorsal skin. In both mouse lines, transgenes were expressed at similar levels in keratinocytes (Fig. 1A and [18], [19]). The wound sites were photographed at the indicated times and the wound area at each time point was compared to that of the original excision (Fig. 1B, C). The wounds of K5-GR and K5-GR-TR healed slower than those of WT (17% and 8%, respectively) at d4. However, at d8, only K5-GR showed delayed wound healing whereas K5-GR-TR wounds were similar to those of WT (Fig. 1C). Immunostaining shows that endogenous GR was mostly localized in the cytoplasm of all epidermal layers and hair follicles (HF) of WT and transgenic mice. In transgenic mice, GR and GR-TR were constitutively nuclear in the basal keratinocytes of the epidermis and HF (Fig. 1D, IH GR; [18], [19]). Histological comparison of the wounds demonstrated that overexpression of both GR and GR-TR in keratinocytes caused an impaired healing response at d4 (Fig. 1D). This delay in healing persisted at d8 in K5-GR mice (Fig. 1D). Wound re-epithelialization was evaluated by measuring the re-epithelialized edges (arrows) relative to those of the original wounds (arrowheads) (Fig. 1D and 1E). In WT at d4, well developed epithelial tongues were observed at both sides of the wound center. At d8, WT wounds were closed although epidermal hyperplasia persisted adjacent to the wound and *de novo* hair follicle formation had not yet occurred (Fig. 1D). In contrast, at d4 the keratinocytes had not begun migrating into the wound bed in K5-GR and K5-GR-TR wounds and the percentage of re-epithelialization relative to WT was greatly reduced (Fig. 1D and 1E). However, the delayed wound healing only persisted at d8 in K5-GR mice whereas K5-GR-TR wounds were closed at that stage similar to WT mice (Fig. 1D and E). Trichrome staining in d8 wounds showed a reduced collagen content in the granulation tissue of K5-GR transgenic mice (Figure 1D, circled). Altogether, these data indicate that keratinocyte-targeted overexpression of GR and GR-TR is sufficient to repress early re-epithelialization. However, only GR was able to reduce collagen deposition at later stages of wound healing.

**Figure 2:**
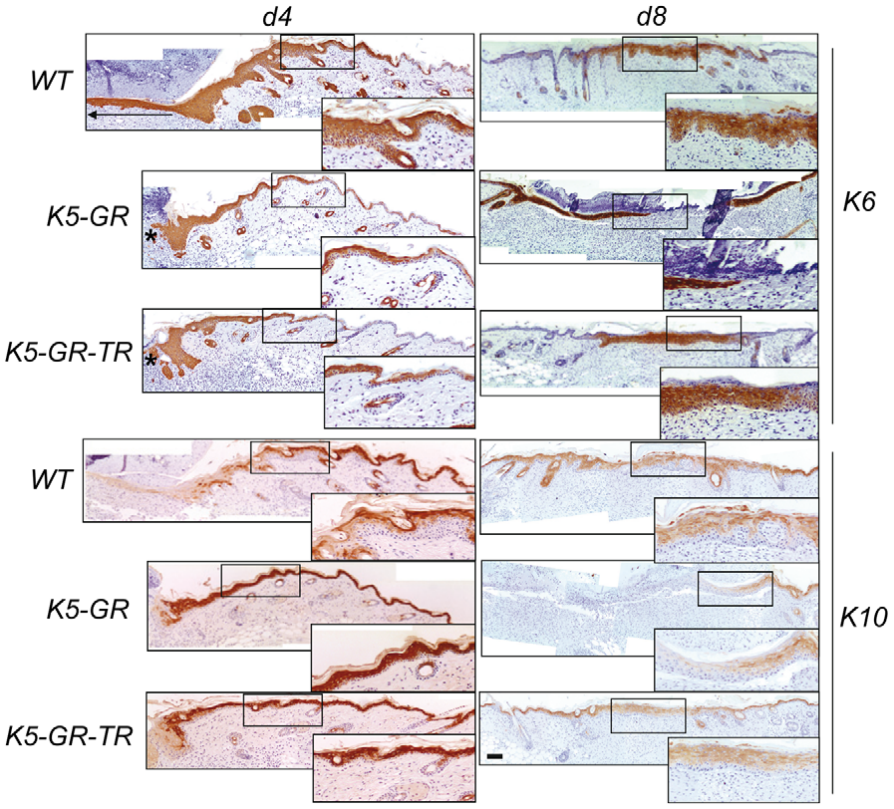
Reduced migration and increased differentiation of keratinocytes in K5-GR wounds relative to K5-GR-TR and WT mice. Skin wounds were collected from WT, K5-GR or K5-GR-TR mice at d4 and d8 to evaluate the expression of K6 and K10 by immunostaining using specific antibodies. Representative results from 5 mice of each genotype are shown. The arrow indicates the migrating tip of the WT wound and the asterisks indicate the impaired keratinocyte migration in the transgenics. Boxed areas are enlarged. Higher magnification of boxed areas is at the lower right of each image. Bar: 100 µm.

Since re-epithelialization depends on proliferation and migration of keratinocytes, we examined these processes in the wounds of K5-GR and K5-GR-TR. We assessed keratinocyte migration by evaluating the architecture of the epithelial tongue and the expression of K6 by immunostaining (Fig. 2, K6). Immunolocalization of the epidermal differentiation marker K10 was assessed in serial sections of skin wounds (Fig. 2, K10). In WT at d4, the tip of migrating keratinocytes stained positive for K6 and negative for K10 (Fig. 2, d4, WT; arrow indicates the migrating tip).

**Figure 3:**
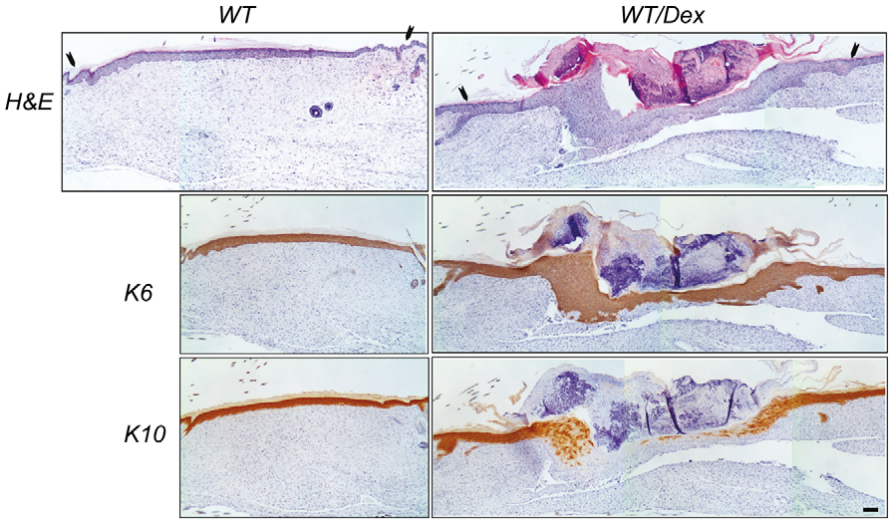
Delayed wound healing response in K5-GR mice is equivalent to that of Dex-treated WT mice. Histological analysis of re-epithelialization of skin wounds in WT mice untreated or treated with Dex (1 mg/kg/day) for 7 d before wounding, at d9 after injury. Arrowheads indicate original wound edges. Immunostaining using anti-K6 and anti-K10 specific antibodies shows the delayed healing in Dex-treated *vs* untreated WT mice (*n* = 6). Representative images of three independent experiments are shown. Bar: 100 µm.

The hyperplasic skin adjacent to the wound also expressed K6 in all epidermal layers, which decreased progressively distal to the wound coincident with increased K10 expression (Fig. 2, d4, WT; see insets). In contrast, wounds of K5-GR and K5-GR-TR only had incipient epithelial tongues (Fig. 2, d4, K6, asterisks), with more restricted K6 staining adjacent to the wound. Contrary to WT, K10 expression was detected continuously throughout the skin of K5-GR and K5-GR-TR mice, with the exception of the tip of the epithelial tongues (Fig. 2, d4, K10). In contrast at d8, only overexpression of GR interfered with keratinocyte migration, as shown by reduced K6 immunostaining in K5-GR wounds relative to WT and K5-GR-TR (Fig. 2, d8). The lack of continuous K10 staining in K5-GR d8 wounds indicated that the restoration of the epidermal barrier was still incomplete. Remarkably, the delayed wound healing of K5-GR mice closely mimicked the healing response of WT mice treated with Dex for 7 d prior to wounding, as shown by K6 and K10 immunostaining (Fig. 3).

**Figure 4:**
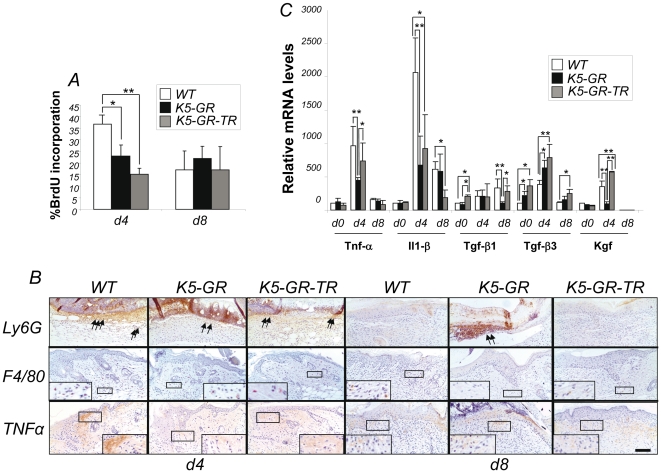
K5-GR and K5-GR-TR mice exhibit reduced proliferation and inflammatory response following injury. A) Keratinocyte proliferation in WT and transgenic wounds. The proliferation rate was quantified by counting BrdU-labeled nuclei in the basal layer of interfollicular epidermal keratinocytes at d4 and d8. Positive nuclei were counted from the BrdU-labeled keratinocyte most proximal to the wound margin to the area at which the BrdU labeling index fell below 5%. The graph illustrates the percentage of positive BrdU keratinocytes *vs* total nuclei. Mean values ± SD are shown (n = 36). Asterisks denote statistically significant differences among genotypes within a given timepoint, as determined by ANOVA (*, p<0.05; **, p<0.01). B) Cutaneous inflammation in WT and transgenic wounds. Immunostaining of WT, K5-GR and K5-GR-TR wounds at d4 and d8 showing granulocytes (Ly6G, arrows), macrophages (F4/80) (inset), and TNF-α (inset). Representative images of three independent experiments are shown. Bar: 50 µm. C) Altered gene expression in transgenic skin wounds. Transcript levels of *Tnf-* α, *Il-1 β*, *Tgf-β1*, *Tgf-β3*, and *Kgf* were determined in the wounds of the indicated genotypes at d4 and d8 after injury by quantitative RT-PCR. Experiments were performed using at least three individuals of each genotype per timepoint. The mRNA levels of WT mice before wounding for each gene were set to 100, and fold-changes related to this basal level. Mean values ± SD are shown. Asterisks denote statistically significant differences among genotypes for each gene at a given timepoint, as determined by ANOVA (*, p<0.05; **, p<0.01).

Proliferation in the wounds was quantified by counting BrdU-labeled nuclei in the basal keratinocyte layer from the BrdU-labeled keratinocyte most proximal to the wound margin to the area at which the BrdU labeling index fell below 5% [20]. There was a statistically significant reduction in keratinocyte proliferation in the wounds of both K5-GR (23%) and K5-GR-TR (15%) mice relative to WT (37%) at d4 after wounding (Fig. 4A). The growth rate paralleled the observed differences in epidermal hyperplasia, which was more pronounced and sustained in the injured skin in WT than in transgenic mice (Fig. 2). At later stages of the wound repair, the rate of keratinocyte proliferation was equivalent in all genotypes (Fig. 4A). Altogether, our results show that overexpression of either GR or GR-TR inhibits keratinocyte proliferation and migration, and induce differentiation, resulting in delayed healing.

It is well known that many of the anti-inflammatory properties of GR reside in its TR function; however, increasing evidence indicates that TA also plays an important role [13], [14]. We examined and compared the immune infiltrate in K5-GR and K5-GR-TR mice during wound healing by immunostaining with specific antibodies for markers of granulocytes (Ly6G), and macrophages (F4/80). Most granulocytes were present within the clot 4 days after wounding, making it difficult to assess cell number. Nonetheless, neutrophil recruitment was reduced in the skin of transgenic animals (Fig. 4B, d4, arrows). At d8, neutrophils were only detected at the wound scab of K5-GR, consistent with the delayed kinetics of healing (Fig. 4B, d8, arrows). Macrophage recruitment in wounded skin was strongly reduced in K5-GR and K5-GR-TR relative to WT (Fig. 4B, d4, see inset for higher magnification). At d8, macrophages were reduced in both transgenics relative to WT, being virtually absent in K5-GR mice (Fig. 4B, d8). We also examined the expression of TNF-α by immunohistochemistry and assessed the staining specificity by using a negative control (secondary antibody only) along with a positive control (a PMA-treated WT mouse skin section), as shown in Fig. S1. We observed reduced levels of this pro-inflammatory cytokine in K5-GR and K5-GR-TR injured skin relative to WT at d4. However, TNF-α down-regulation was more evident in K5-GR than in K5-GR-TR. At d8, we detected similar weak expression of this cytokine in all genotypes (Fig. 4B). Altogether, our data indicate that at later stages, the healing response of K5-GR-TR mice was more similar to WT than to K5-GR.

Given that the many of the growth factors and cytokines playing a major role in wound healing are repressed by GCs, we examined the expression of several of these genes by quantitative RT-PCR. We checked gene expression of *Tnf-α*, *Il-1β*, *Tgf-β*1, *Tgf-β*3 and *Kgf* before and after wounding (Fig. 4C). In uninjured skin of all genotypes, there were no statistically significant changes in gene expression with the exception of *Tgf-β*1 and *Tgf-β*3. *Tgf-β*1 was slightly increased in K5-GR-TR skin relative to WT and K5-GR mice whereas *Tgf-β*3 was up-regulated in both transgenics relative to WT.

In WT wounds, the expression of all genes analyzed was induced at d4. At d8 mRNA levels of all genes declined, with the exception of *Tgf-β*1. In K5-GR d4 wounds, the expression of *Tnf-α*, *Il-1β* and *Kgf* was repressed in comparison to WT, whereas *Tgf-β*3 mRNA was up-regulated. At d8, transcript levels were similar in K5-GR and WT mice for all the genes analyzed except for *Tgf-β*1, which was down-regulated in K5-GR wounds. Importantly, we observed differences in the amplitude and kinetics of the response in GR and GR-TR transgenic mice. In d4 K5-GR-TR wounds, *Il-1β* was repressed to a lesser extent than in K5-GR wounds. However, unlike in K5-GR mice, the repression of *Il-1β* continued at d8. Importantly, *Tnf-α*, *Kgf* and *Tgf-β*1 mRNA levels were not repressed in K5-GR-TR wounds and were similar to those of WT.

**Figure 5:**
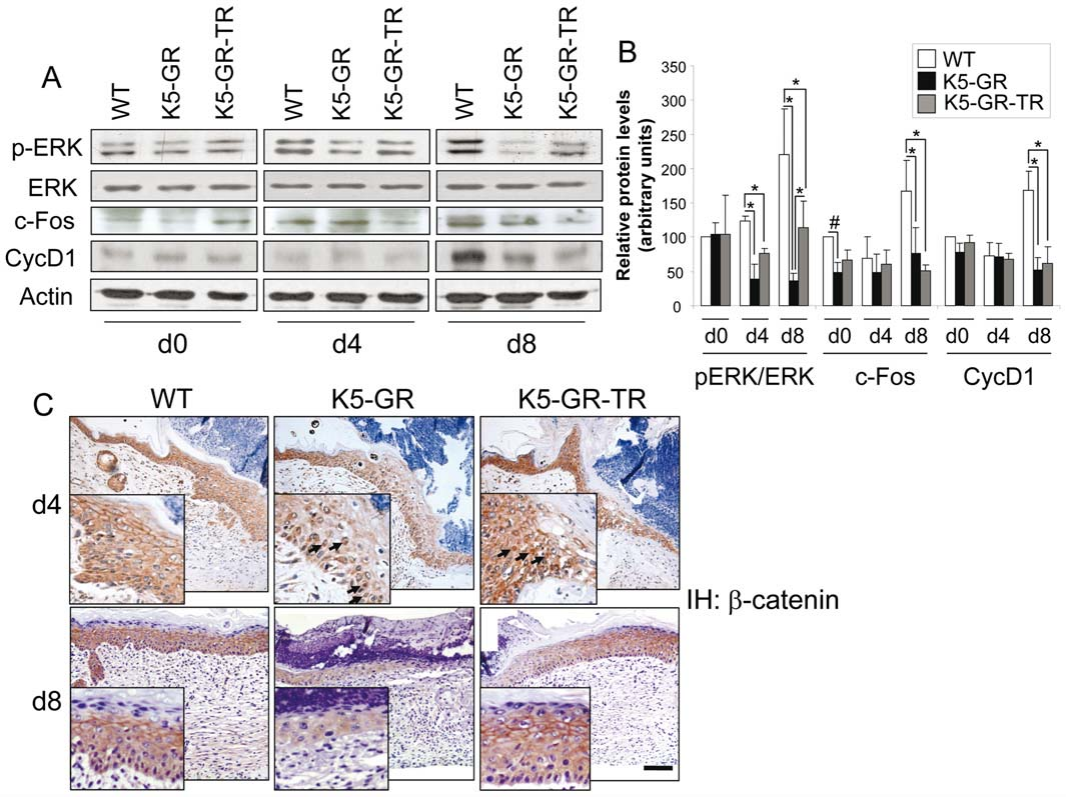
K5-GR and K5-GR-TR wounds show reduced ERK activity. A) Changes in protein expression after wounding. Total skin protein was extracted from d4 and d8 wounds of WT, K5-GR and K5-GR-TR mice and subjected to immunoblotting using the indicated specific antibodies (p-ERK, ERK, c-Fos, and cyclin D1). Actin was used as a loading control. Panels are representative of the relative expression levels of the indicated proteins for each condition. B) Quantitation of the experiments performed using at least three individuals of each genotype per timepoint. Protein levels were normalized to actin. The graph shows the p-ERK/ERK ratio as well as c-Fos and cyclin D1 protein levels in K5-GR, K5-GR-TR and WT wounds. The protein levels of WT mice before wounding were set to 100, and fold-induction related to this basal level. Mean values ±SD are shown. Asterisks denote statistically significant differences at each timepoint among all genotypes, as determined by ANOVA (*, p<0.05; **, p<0.01). C) Immunostaining of sections of WT, K5-GR and K5-GR-TR wounds at d4 and d8 showing the expression pattern of β-catenin. Arrows denote the nuclear localization of β-catenin in transgenic keratinocytes. Representative images of each condition are shown. Bar: 50 µm.

The antagonism between GR and the MAPK ERK has been reported in several cell types, including keratinocytes [1], [21], [22]. We assessed whether impaired ERK activity was involved in the wound delay elicited by GR and GR-TR *in vivo* by analyzing the relative levels of ERK and p-ERK and downstream targets c-Fos and cyclin D1 by immunoblotting. The ratio of phosphorylated (p)-ERK/ERK was significantly increased in WT mice at d8 following wounding whereas both transgenic mice showed statistically significant decrease in ERK activity relative to WT wounds at d4 and d8 (Fig. 5A, B). Remarkably, the decrease in phospho-ERK at d8 was more pronounced in K5-GR than in K5-GR-TR mice. Following ERK activation, c-Fos and cyclin D1 were induced in WT skin at d8 upon wounding (Fig. 5A, B). In contrast, c-Fos and cyclin D1 were significantly down-regulated relative to WT in both transgenics to the same extent at this timepoint.

Previous reports have described a cross-talk between GR and β-catenin in wound responses [23]. We thus checked whether K5-GR and K5-GR-TR mice have altered expression and/or localization of β-catenin upon wounding. In d4 WT wounds, β-catenin was found at the membrane of suprabasal keratinocytes and in the cytoplasm of more basal epidermal layers (Fig. 5C). In contrast, we detected β-catenin mostly in the cytoplasm of epidermal keratinocytes but also in some nuclei of the transgenics (Fig. 5C, d4). At d8 after wounding, we did not find differences in β-catenin localization among all genotypes. However, β-catenin expression levels were decreased in K5-GR wounds relative to the other genotypes (Fig. 5C, d8).

**Figure 6:**
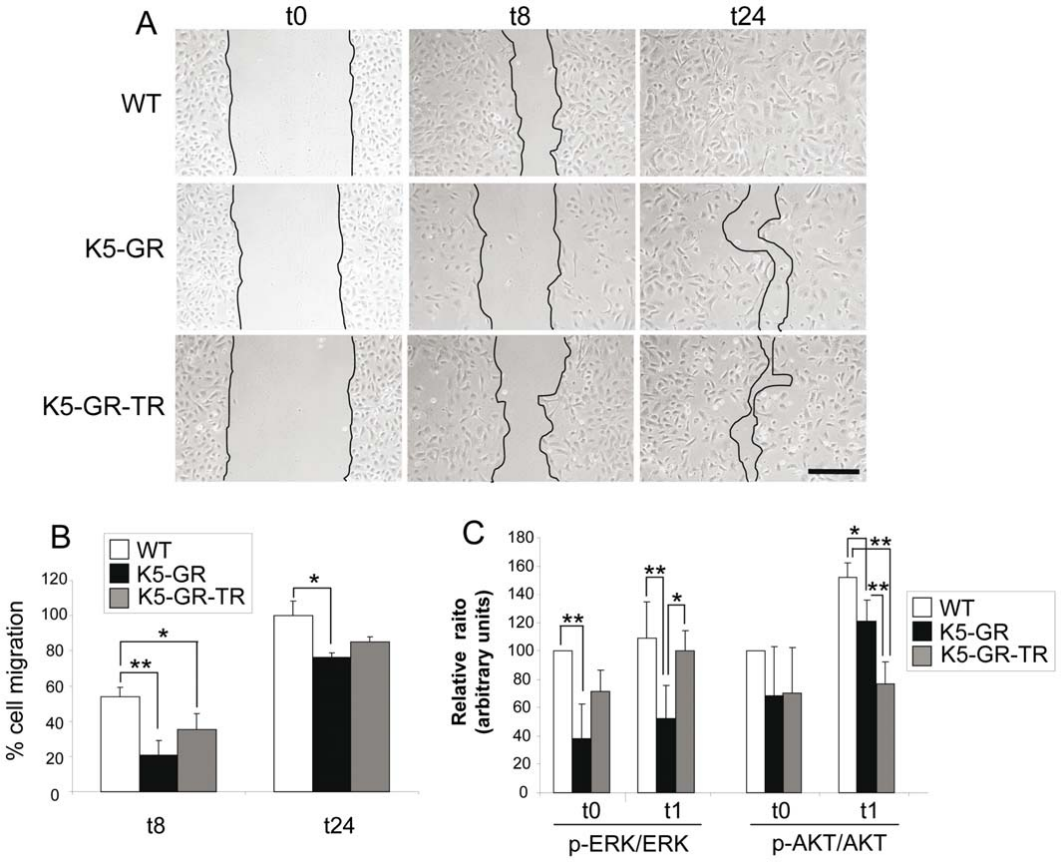
GR and GR-TR delay keratinocyte migration in *in vitro* wound scratch assays. A) *In vitro* migration of K5-GR and K5-GR-TR primary keratinocytes isolated from WT, K5-GR and K5-GR-TR were evaluated for *in vitro* wound closure at 8 h and 24 h following scratching. Phase contrast photographs are representative of four independent experiments. Bar: 100 µm. B) The graph shows the average of wound closure ± SD. Statistically significance was determined by ANOVA (*, p<0.05; **, p<0.01). The percentage of cell migration was estimated as described in Materials and Methods. C) Quantitation of the p-ERK/ERK and p-AKT/AKT ratios in WT, K5-GR and K5-GR-TR keratinocytes before (t0) and after (t1) scratch wounding. Protein levels were determined by immunoblotting using specific antibodies against the total and phosphorylated proteins, and then normalized to actin. Mean values ± SD are shown. Asterisks denote statistically significant differences, as determined by ANOVA (*, p<0.05; **, p<0.005).

To further evaluate the keratinocyte-specific contribution of GR and GR-TR in delayed healing, we performed *wound scratch* assays in primary cultured keratinocytes obtained from K5-GR, K5-GR-TR and WT mice and assessed cell migration (Fig. 6A, B). At 8 h after scratching, we found a significant reduction of keratinocyte migration in both K5-GR and K5-GR-TR cultures relative to WT (20% and 35%, respectively, in comparison to 55%). This trend was still apparent at 24 h (77% and 84% in K5-GR and K5-GR-TR cells, relative to 100% in WT cells); however, the delay in migration was only statistically significant in K5-GR keratinocytes relative to WT.

We next assessed the activity of ERK and AKT by immunoblotting shortly (1 h) after scratch wounding transgenic and WT keratinocytes (Fig. 6C). The p-ERK/ERK ratio was significantly diminished in K5-GR keratinocytes relative to WT, before and after the scratch. In contrast, ERK activity in K5-GR-TR keratinocytes was more similar to that observed in WT. Statistically significant increases in AKT activity following *in vitro* wounding were only observed in WT and K5-GR keratinocytes (Fig. 6C). At t1, the p-AKT/AKT ratio was reduced in K5-GR and K5-GR-TR relative to WT and remarkably, the differences between the two transgenic lines were also statistically significant (Fig. 6C). Overall, our data suggest that distinct patterns of ERK and AKT inhibition mediated by GR and GR-TR in keratinocytes may contribute to the differential wound responses of each transgenic mouse line.

## Discussion

Prolonged GC therapy in human patients is often accompanied by adverse reactions including skin atrophy and delayed wound healing [2]. It has been also reported that psychological stress, likely through the increased production of endogenous GCs, disturbs epidermal barrier competence [24]. Previous efforts to identify the mechanisms responsible for the adverse actions mediated by GR in wound repair relied on the use of synthetic ligands for GR either *in vivo* or in cultured keratinocytes [2], [23]. In this work, we have investigated the effects of keratinocyte-specific expression of the wild-type and mutant forms of GR in wound repair. In both mouse models, the keratinocyte-targeted expression of either GR or GR-TR resulted in nuclear localization of the transgenes, as shown by immunolocalization studies *in vivo* (Fig. 1C; [18], [19]. Remarkably, the phenotype of K5-GR and K5-GR-TR mice recapitulates the skin atrophy and sporadic alopecia observed after prolonged treatments with GC analogs in human patients as both transgenic mice featured epidermal thinning as well as diminished number of hair follicles in the adult age [2], [18], [25]. These alterations were exclusively due to the transgenes and not related to changes in the endogenous circulating GC levels, as plasma corticosterone levels were unchanged in the transgenics relative to WT mice (data not shown). However, the existence of extra-adrenal additional sources of GC synthesis, including the skin [26], needs to be taken into account. Recent work has shown that epidermal keratinocytes can synthesize *de novo* cortisol, as well as other steroid metabolizing enzymes, both *in vitro* and *in vivo* [27]. Furthermore, it was shown that IL-1β synthesis, one of the first events occurring in keratinocytes upon injury, triggers the local production of cortisol. Conversely, augmented IL-1β during wound healing resulted in the inhibition of cortisol synthesis. This highlights the relevance of locally produced GCs and likely represents a feed-back mechanism to modulate GC-mediated signaling and mitigate the initial pro-inflammatory responses [27].

There were statistically significant delays in transgenic wound closure relative to WT. In fact, the slowed healing elicited by the keratinocyte-targeted overexpression of GR in K5-GR mice was very similar to that observed in WT mice that were Dex-treated prior to wounding (Fig. 3). This implies that at least some of the side-effects associated with GC treatment, such as delayed wound healing, are mediated through keratinocyte autonomous GR actions. Overall, our results highlight that K5-GR and K5-GR-TR mice are valuable tools to address the molecular mechanisms underlying keratinocyte-specific GR effects in skin homeostasis.

It is known that several pro-inflammatory cytokines such as IL-1α, IL-1β, IL-6, and TNF-α are typically up-regulated during the inflammatory phase of wound healing, contributing to normal repair [15], [16]. Among these, IL-1β plays a key role in epidermal injury, being one of the first signals activated in keratinocytes. The expression of these cytokines as well as the termination of the inflammatory response through induction of anti-inflammatory cytokines such as IL-10 must be spatio-temporally coordinated [16]. *TNF-α* and *IL-1β* mRNA were induced in WT mice at d4, consistent with the literature (Fig. 4). However, in d4 wounds, the transcript levels of *TNF-α* were significantly diminished in K5-GR as compared to WT and K5-GR-TR mice. The decrease in induction of *IL-1β* was pronounced in both transgenic mice relative to WT at d4; however, at d8 only K5-GR-TR showed a significant decrease in *IL-1β* mRNA. These changes are in agreement with the strong reduction of several pro-inflammatory cytokines including IL-1β reported in Dex-treated mice as well as in diabetic *db/db* mice [17]. Our previous work illustrated that the mechanism(s) used by GR for TR in keratinocytes is gene-specific, as GR-TR efficiently repressed *IL-1β* and *Mmp-3* transcription (to a similar extent as GR) whereas it only weakly repressed that of *IL-6* and *TNF-α* [18].

As macrophages and neutrophils are major sources of IL-1β, the changes in expression in transgenic wounds may be due to either reduced recruitment of inflammatory cells and/or direct repression of IL-1β by GR and GR-TR in keratinocytes. Our immunodetection data showing clear reductions in the dermal inflammatory infiltrate in both transgenics relative to WT correlate well with RT-PCR results (Fig. 4), suggesting that both mechanisms likely contribute to IL-1β repression. These data fit with previous work in GR^dim/dim^ mice showing that endogenous GCs weakly slow early healing in comparison to the drastic effects of exogenous GCs [28]. In these experiments, GR^dim/dim^ mice had increased levels of IL-1β mRNA and minor changes in IL-6 mRNA 1 d after wounding [28]. Our data show that the constitutive expression of GR and GR-TR restricted to keratinocytes has a profound impact on wound healing, similar to the effects of exogenous GCs (Fig. 3).

In agreement with the role of several growth factors such as KGF, TGF-β1 and -β3 in normal repair, we detected their induction in WT upon wounding (Fig. 4C). Previous findings suggested that impaired expression of TGF-βs was associated with the wound healing defects associated with GC treatment and diabetes in rodents [15]. We detected TGF-β1 and KGF repression in K5-GR but not in K5-GR-TR mice. However, TGF-β3 was induced in the wounds of both transgenic mice. Overall, our data suggest that the different healing kinetics documented in K5-GR *vs* K5-GR-TR mice may be accounted for by the distinct patterns in gene expression regulated by each transgene (Fig. 4C).

It is known that the GR-TR mutant used in this study is not completely TA-deficient but a mutant with impaired GR-mediated transcription [12]. Nonetheless, our results support the idea that the TA function of GR is linked to the unwanted side-effects associated with this receptor. The re-epithelialization rate at d4 was greatly reduced in both K5-GR and K5-GR-TR mice relative to WT as a consequence of the inhibition of keratinocyte proliferation and migration (Figs. 2 and 4). In addition, both GR and GR-TR transgenes were able to inhibit migration of cultured keratinocytes at an early timepoint, similar to the reported effects of Dex treatment [22], [29]. These results suggest that the effects of GR on keratinocyte migration are mediated by its TR function. Our data are compatible with wound scratch experiments performed in cultured GR^dim/dim^ fibroblasts showing increased proliferation and migration relative to WT [28]. On the other hand, GR and GR-TR had different effects on tissue remodeling given the reduction in dermal collagen deposition in K5-GR relative to K5-GR-TR mice (Fig. 1), consistent with the known effects of GR on collagen synthesis and deposition [17]. These results are in agreement with data reported in GR^dim/dim^ wounds, which exhibited an extended granulation tissue at d5 after injury without changes in collagen or tenascin-C [28].

It has been reported that keratins K6 and K16, which play an important role in keratinocyte migration, are transcriptionally down-regulated by GR through different mechanisms [30]. These include the interference of GR with the MAPK signaling pathway, as previously shown in transgenic mice with GR gain- and loss-of-function [21], [22]. In WT mice, *k6* mRNA is induced upon wounding through AP-1 sites at its promoter. GR represses this transcript by negatively interfering with AP-1 as well as through direct binding of four GR monomers to nGREs in the *k6* promoter [31]. Another proposed mechanism for K6 repression involves the nuclear localization of β-catenin and its stabilization upon GR activation, leading to c-myc induction in chronic wounds [23]. The activation of β-catenin/c-*myc* would contribute to delayed wound healing by inhibiting migration and altering keratinocyte differentiation. In agreement with this, we have detected nuclear β-catenin in K5-GR and K5-GR-TR keratinocytes *in vivo* at early stages of wound healing (Fig. 5). The cross-talk between GR and β-catenin, which interact *in vitro*, suggests a relevant role of Wnt-mediated signalling in the therapeutical actions of GCs [32].

It is known that ERK phosphorylation followed by its translocation to the nucleus is critical for the G1 to S phase transition, leading to cell proliferation [33]. In addition, the activation of ERK leads to induction of cyclin D1 [33]. In other cell types, it has been reported that ligand-activated GR suppresses phosphorylation of ERK1 and cyclin D1 expression, resulting in cell growth inhibition [34]. Our findings are in agreement with this and suggest that GR/ERK interference and the down-regulation of c-Fos and cyclin D1 contributes to the impaired healing response of both K5-GR and K5-GR-TR mice. Furthermore, the diminished transcript levels of several genes that are AP-1 targets including *Tnf-α*, *Il-1β*, and *Tgf-β1* support a role of reduced AP-1 activity in delayed wound healing (Figs. 4 and 5). In summary, through study of the K5-GR and K5-GR-TR mouse models, we have shown that the TR function of GR is sufficient for inhibition of the early stages of wound healing, likely through inhibition of MAPK signaling and AP-1 dependent transcription. In contrast, the TA function of GR is required for repression of later stages of wound closure likely due to suppression of tissue remodelling. The decreased efficiency of inhibition of ERK phosphorylation exerted by GR-TR relative to GR both *in vivo* and *in vitro* may allow for more rapid wound closure (Figs. 5 and 6). In this regard, the differential inhibition of ERK activity between the transgenic lines may explain the observed differences in their wound healing kinetics.

Given the high prevalence of skin diseases and the wide use of GCs for their treatment, the selective modulation of GR function is a major therapeutic objective. The development of non-steroidal selective GR agonists or SEGRAs in the recent years is based on the ability of these compounds to dissociate the TA *vs* TR properties of GR [35]. In this regard, our observations that epidermal expression of the GR-TR mutant is less deleterious to wound healing than WT GR suggest that the use of SEGRA compounds mimicking GR-TR could be beneficial. The selective activation of GR-TR should elicit a partial inhibition of ERK and minor repression of pro-inflammatory cytokines and growth factors relative to activation of both TA and TR functions, thus contributing to improved re-epithelialization and remodeling. The use of SEGRA compounds mimicking GR-TR could achieve a better benefit/risk ratio by minimizing delayed wound healing, one of the most common adverse side-effects associated to prolonged GC treatment.

## Materials and Methods

### Ethics Statement

All mice were handled in accordance with the current Spanish and European regulations which govern research with animals (Real Decreto 1201/2005, B.O.E. #252, 10 of October, 2005 and Convenio Europeo 1-2-3 del 18/3/1986), and approved by the Ethic Committee of the Spanish Research Agency Consejo Superior de Investigaciones Científicas CSIC. All projects granted by the Spanish Ministery need to be approved by the institution's Ethic Committee and this is granted by the approval ID of the project SAF2008-00540.

### Animals and wounding experiments

K5-GR and K5-GR-TR mice have been previously reported [18], [19]. Both transgenic lines were generated in a C57Bl6/DBA//F2 genetic background and maintained by backcrosses with C57Bl6/DBA//F1 mice (Harlan). Cutaneous wounding was performed in 12 week old female mice of the indicated genotypes, using WT littermates (C57Bl6/DBA//F2) as controls. At least 16 mice of each genotype were used for each timepoint. Where indicated, WT mice (n = 6) were treated either with vehicle or Dex (Sigma, St. Louis, MO, 1 mg/kg body weight, injected subcutaneously) daily for 7 d before wounding. The treatment was continued for another 9 d after wounding and then, mouse dorsal skin was shaved and wounds were collected.

Full-thickness wounds were performed by using 6 mm diameter punches (Biopsy punch, Stiefel). The percentage of wound closure was quantitated at different timepoints (d0, d4, d8) by tracing the perimeter of the healing wound onto saran wrap, as previously described [36]. Following sacrifice, cutaneous wounds were collected for histopathological analysis, and isolation of RNA and proteins.

For preparation of mouse primary keratinocytes for wound scratch assays, skin from newborn mice of each genotype (n = 21) was excised and processed, as previously described [21]. Following overnight culture in EMEM 1% FBS, confluent dishes of cells were treated with mitomycin C (Sigma, 10 µg/ml) for 1 h before wounding. Scratch wounds were made in cell monolayers with sterile yellow pipette tips. Following wounding, cultures were photographed at 8 h and 24 h to quantitate keratinocyte migration [22]. The area that remained uncovered by the cells was quantitated and divided by the area of the original scratch to calculate the percentage of cell migration into the wound. (Adobe Acrobat 8 Professional). Six images were analyzed per condition and time-point.

### Antibodies

The antibodies used included rabbit polyclonal antibodies against GR (sc-1004), β-catenin (sc-7199), ERK (sc-154), c-Fos (sc-52), and Akt (sc-1619) from Santa Cruz Biotechnology, Inc., (Santa Cruz, CA). Antibodies against keratin K6 (PRB-169P) and K10 (PRB-159P) were from Covance (Babco, Berkeley, CA), anti-p-ERK (Thr202/Tyr204) (#4376) and p-Akt (ser-473) (#9271) were purchased from Cell Signaling (Cell Signaling Technology Inc., Beverley, MA). F4/80 antibody was from AbD serotec (MorphoSys, Oxford, UK), Ly6G from BD Pharmingen, anti-TNF-α from Calbiochem, and anti-actin (A2066) was from Sigma. Secondary peroxidase-conjugated anti-rabbit and anti-goat antibodies were from Amersham (Aylesbury, UK) and secondary peroxidase-conjugated anti-mouse antibody was from Jackson ImmunoResearch (Jackson ImmunoResearch Laboratories, Inc. West Grove, PA). Secondary biotin-conjugated anti-rabbit or anti-mouse antibodies were from Jackson ImmunoResearch.

### Histological and Immunohistochemical analysis

Cutaneous wounds were collected at the indicated timepoints, fixed in 70% ethanol, bisected through the center of the wound and both halves of the wound were embedded in paraffin to obtain 4 µm-thick sections, and then stained with H&E.

To compare the histopathology of the lesions the sections were selected with the largest diameter along the center of the wound. The width between epithelial edges and between wound edges (dermis edges) was measured using photographs of the wound sections and Adobe Acrobat 8 Professional. The re-epithelialization rates were then calculated by using the formula: re-epithelialization = ([distance between opposing dermis edges – distance between opposing epithelium edges]/distance between opposing dermis edges)×100%, as previously described [20].

For immunohistochemistry, paraffin sections were blocked with 5% fetal bovine serum, and then incubated with primary antibody for at least 1 h. Slides were washed three times with PBS, and then incubated with conjugated secondary antibodies for 1 h. Finally, the reaction was visualized with the Avidin-Biotin- Complex (ABC) kit from DAKO (Vectastain Elite, Vector Laboratories, Inc, Burlingame, CA) using diamino-benzidine as chromogenic substrate for peroxidase. Trichrome staining was performed by using a commercial kit from Sigma (HT15-1KT). Slides were mounted and analyzed by light microscopy (Leica DM RXA2), and images were taken at the indicated magnification.

### *In vivo* epithelial BrdU labeling

Epithelial cell proliferation was measured by i.p. injection of BrdU (130 µg/g of body weight, Roche) into mice 1 h before sacrifice. BrdU incorporation was detected by immunohistochemistry of paraffin-embedded sections using a mouse anti-BrdU monoclonal antibody (biotest, Roche) followed by hematoxylin counterstaining. The number of BrdU-positive cells and the number of total cells was determined per 200 µm of interfollicular epithelium in each section. Experiments were performed at least in five individuals of each genotype and differences were assessed by using the ANOVA test.

### RNA preparation and quantitative RT-PCR

Total RNA was isolated from wounds of transgenic and control littermates (five animals of each genotype) by using Trizol reagent (Invitrogen, Molecular Probes, Eugene, Oregon), following manufacturer's recommendations. Reverse transcription was performed by using 1 µg of RNA and oligo-dT (Fermentas Inc., Burlington, Canada) followed by quantitative PCR using specific oligonucleotides for the genes indicated and FastStart Universal SYBR Green Master ROX (Roche, Indianapolis, IN) in an Applied Biosystems 7500 Fast real time PCR system (Applied Biosystems, Foster City, CA). Oligonucleotide sequences are as follows: *TNF-α* (forward: 5′-ATG AGC ACA GAA AGC ATG ATC-3′; reverse: 5′-TAC AGG CTT GTC ACT CGA ATT-3′), *IL-1β* (forward: 5′-ATG GCA ACT GTT CCT GAA CTC ACC T-3′; reverse: 5′-CAG GAC AGG TAT AGA TTC TTT CCT TT-3′), *TGF-β*1 (forward: 5′-CTT CAG CTC CAC AGA GAA GAA CTG-3′; reverse: 5′-CAC GAT CAT GTT GCA CAC TGC TCC-3′), *TGF-β*3 (forward: 5′-TGG CGG AGC ACA ATG AAC TGG-3′; reverse: 5′-CCT TTG AAT TTG ATT TCC ATC-3′), and *Kgf* (forward: 5′-TGC AGC AAC TGG CCT TGT CAC-3′; reverse, 5′-TCC AAC TGC CAC GGT CCT GAT-3′). At least five individual were used per genotype and timepoint, and technical triplicates were assessed to calculate the mean value ± SD. Statistically significant changes were determined by performing an ANOVA test.

### Immunoblotting

Whole-cell protein extracts were prepared, boiled in Laemmli buffer, separated on 10% SDS-PAGE, and then transferred to nitrocellulose filters (Hybond ECL; Amersham), as previously described [21]. Membranes were analyzed using the enhanced chemiluminescence method (ECL, Amersham), according to the manufacturer's recommendations. The membranes were also stained with Ponceau S (Sigma) to verify equal protein loading and transfer. Actin was used as a loading control. Specific bands were scanned by using HP Scanjet G4010, quantitated with ImageJ (National Institutes of Health), normalized to actin, and plotted on a bar graph. Values for WT samples (d0 after wounding) were arbitrarily set as 100, and the other experimental groups expressed as relative to WT. Experiments were performed in at least three individuals of each genotype, and differences were assessed by using ANOVA test, with statistical significance when *p*<0.05.

## Supporting Information

Figure S1 Specificity of TNF-α immunostaining. The dorsal skin of adult WT mice was topically treated with either vehicle (untreated) or PMA (8 µg) for 2 weeks, and immunostaining using a secondary antibody only (left) or TNF-α antibody (right) was performed. Representative images of three independent experiments are shown.
(TIF)
