# Supplementary figures and images for: Keratinocyte-Targeted Overexpression of the Glucocorticoid Receptor Delays Cutaneous Wound Healing
Source: PLoS One. 2012 Jan 3;7(1):e29701. doi: 10.1371/journal.pone.0029701 (PMC3250471; doi:10.1371/journal.pone.0029701)

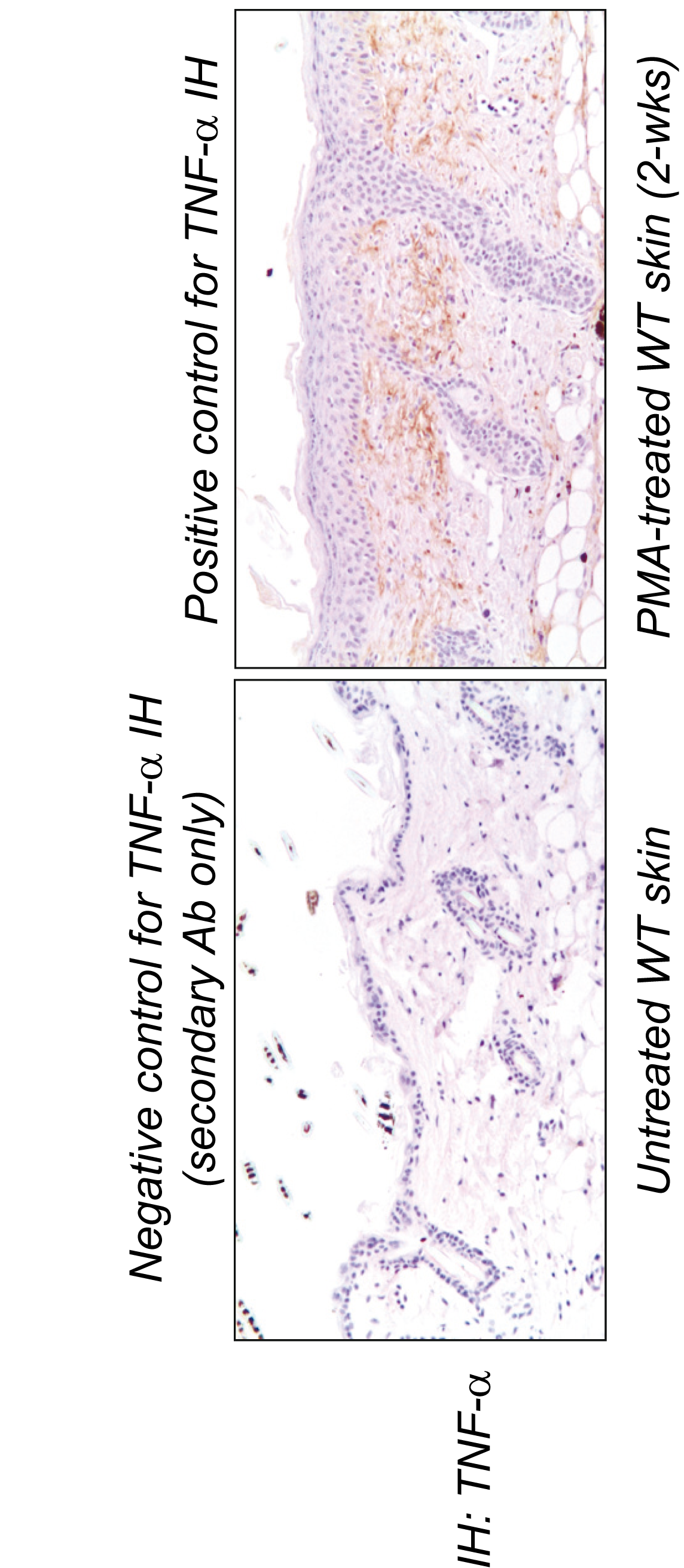

Supplement: Figure S1 — Specificity of TNF-α immunostaining. The dorsal skin of adult WT mice was topically treated with either vehicle (untreated) or PMA (8 µg) for 2 weeks, and immunostaining using a secondary antibody only (left) or TNF-α antibody (right) was performed. Representative images of three independent experiments are shown. (TIF) [file pone.0029701.s001.tif]
